# Comparing Adult Males and Females in the United States to Examine the Association between Body Mass Index and Frequent Mental Distress: An Analysis of Data from BRFSS 2011

**DOI:** 10.1155/2013/230928

**Published:** 2013-11-14

**Authors:** Soumyadeep Mukherjee

**Affiliations:** Department of Epidemiology, Robert Stempel College of Public Health & Social Work, Florida International University, 11200 University Park, AHC 595-1, 11200 SW 8th Street, Miami, FI 33199, USA

## Abstract

*Background*. There is conflicting evidence regarding the association of body mass index (BMI) with mental distress. Studies have focused on different dimensions of mental health and used different definitions and many of them have not controlled for confounding factors. The aim of this study was to examine the relationship between frequent mental distress (FMD) and BMI among adults in the United States, with special emphasis on gender differences. *Methods*. Data from the Behavioral Risk Factor Surveillance System (BRFSS) for the year 2011 were used in logistic regression models to predict FMD, defined as having 14 or more days of poor mental health in the previous month. Sociodemographic factors, tobacco and alcohol use, diet and physical activity, and number of chronic diseases were controlled for. *Results*. 11.95% (*n* = 53,715) of the participants with valid responses (*n* = 496,702) had FMD. The adjusted ORs of having FMD among underweight, overweight, and obese females were 1.13 (95% CI: 1.10, 1.60), 1.10 (95% CI: 1.03, 1.19), and 1.21 (95% CI: 1.13, 1.31), respectively, but they were not statistically significant for males. *Conclusions*. These findings suggest a relationship between BMI and FMD, independent of other variables. It may be useful to explore longitudinal trend in this association.

## 1. Introduction

Mental illness is a term used to describe “health conditions that are characterized by alterations in thinking, mood, or behavior (or some combination thereof) associated with distress and/or impaired functioning” [1]. In any particular year, approximately 25% of all adults (people aged 18 and above) in the United States (U.S.) have mental illness with an economic burden of about 300 billion U.S. dollars [2]. Almost half of all U.S. adults will be affected by at least one mental illness during their lifetime [3]. One of the ways to estimate the burden of mental illness is by looking at frequent mental distress (FMD), that is, having 14 or more days of poor mental health in the previous 30 days [4]. Poor mental health, which includes stress, depression, and problems with emotions, is ascertained by the subjective appraisal of one's own mental status [5]. In addition to its impact on quality of life [6], mental distress is associated with suicidal ideation and attempts [7] and chronic conditions, such as diabetes, cardiovascular disease, and cancer [8]. Harmful behaviors, such as physical inactivity, engaging in risky sexual behaviors, violence, and substance use, are related to mental distress [9–11]. A less certain area is the association of body mass index (BMI) with mental health; while some studies have reported an association [12, 13], others did not find any such evidence [14, 15]. BMI, calculated as weight (kg)/[height (m)]^2^, is the basis for classifying adults as underweight (BMI: below 18.5), normal (BMI: 18.5–24.9), overweight (BMI: 25.0–29.9), and obese (BMI: 30.0, and above) [16]. 

Although precise mechanisms are not clear [17], common underlying genetic factors [18] and biophysiological mechanisms [19] are implicated behind the relationship of obesity with poor mental health. Stigma and discrimination associated with being obese and overweight can lead to mental health consequences [20, 21]. Body image, which is the psychological experience of the appearance and function of one's own body and an aspect of the person's mental representation of himself/herself [22], partially explains the relationship between obesity and mental distress [23]. Self-Dissatisfaction with weight is more common among women than men [24] and especially among white women compared with black women [25]. Sociodemographic factors, physical activity, and nutrient intake might influence the relationship between BMI and psychological distress [26, 27].

In 2007, obese men and overweight or obese women in the United States had a significantly higher prevalence of serious psychological distress, compared with people having normal BMI [28]. Serious psychological distress, unlike FMD, is an estimate of serious mental illness and has a lower prevalence [29, 30]. Certain subgroups within the obese population [31], those seeking treatment to lose weight [32, 33] and binge-eaters, might be more prone to psychological problems. Some studies have not found any association between BMI and mental distress; some others have reported that mental distress decreases with an increase in BMI [14, 34–36].

Fewer studies have investigated the relationship between being underweight and mental health, with evidence supporting [28, 37] as well as refuting [38]. While investigating the association of low BMI and mental distress, confounding factors like being unmarried, unemployed, or disabled should be considered [39–41]. 

The evidence is inconclusive regarding an association between BMI and mental distress. Different studies have focused on different dimensions of mental health, used different definitions of mental illness, and studied different populations and many of them have not controlled for all the potential confounding factors. The primary aim of this study was to explore the association between FMD and BMI in a representative sample of U.S. adults included in the Behavioral Risk Factor Surveillance System (BRFSS) dataset of 2011. The secondary aim was to examine whether this association differed between males and females. The hypothesis was that people who do not have a normal BMI are more likely to suffer from FMD than those in the normal BMI category, even after adjusting for all the covariates.

## 2. Methods

### 2.1. Study Population

This study is based on the analysis of 2011 BRFSS results. BRFSS, the principal health-related telephone survey among a representative sample of U.S. residents aged 18 years and above, collects information about the respondents' risk behaviors and events affecting health, chronic health conditions, and use of preventive services. A total of more than 506,000 interviews were conducted in 2011 [42].

### 2.2. Measuring FMD

The BRFSS questionnaire has an item asking the respondent to report the number of days his/her mental health was not good in the previous 30 days [43]. All the participants who had 14 or more days of “not good” mental health in the previous month were categorized as having FMD [4] and the rest were categorized as not having FMD. 

### 2.3. Independent Variables

The variable categorizing individuals based on their BMI was the main predictor for this analysis. Age categories in years, gender, race/ethnicity, level of education completed, employment status, income level, and marital status were the sociodemographic covariates. Tobacco and alcohol use were considered. Other lifestyle factors included were number of healthy food items and physical activity. The number of obesity related chronic conditions was taken into account, depending on whether a health professional ever told them of having high blood pressure, high cholesterol, heart disease, stroke, asthma, diabetes, or arthritis. 

### 2.4. Statistical Analyses

The respondents who answered as “don't know/not sure,” “refused,” or had missing responses at random were excluded from the analyses. Women who reported being told of having high blood pressure and/or diabetes only during their pregnancy were also excluded. Logistic regression analysis was used to investigate the association of FMD with BMI and other covariates. Each of the independent variables was separately used to predict FMD. This was followed by a multivariable model, where all the variables were simultaneously introduced. Finally, separate multivariable analyses were performed after stratifying for gender. All the independent variables were categorical, except the number of healthy food items (0–4) and number of ever-diagnosed chronic conditions (0–6), which were treated as continuous in multivariable analysis. All analyses were performed using SAS statistical software package, version 9.3 (SAS Institute Inc., Cary, North Carolina). Adjustments were made for the sampling design and for the raking procedure used to assign respondent weights [44] by using “proc survey” procedures in SAS [45].

## 3. Results 

### 3.1. Characteristics of the Sample

There were 198812 males (weighted percentage: 48.73%, 95% CI: 48.44%, 49.02%) and 307655 females (weighted percent: 51.27%, 95% CI: 50.98%, 51.56%) in the BRFSS 2011 sample (Table 1). Most of the respondents were non-Hispanic Whites (weighted percent: 66.42%). 1.87%, 35.85%, and 27.42% of the sample were underweight, overweight, and obese, respectively. Overall prevalence of frequent mental distress (FMD) was 10.8%, with a weighted percentage of 11.95% (95% CI: 11.75%, 12.14%). 

### 3.2. Bivariate Analysis Results

From Table 2, females were 1.4 times likely to report FMD compared to males (OR: 1.39, 95% CI: 1.34, 1.44). Non-Hispanic Asians had lower odds (OR: 0.50, 95% CI: 0.42, 0.60), but people of all the other races had significantly higher odds of reporting FMD compared to non-Hispanic Whites. Participants who graduated from high school and those who had higher education were significantly less likely to suffer from FMD. Compared to people who were employed for wages, participants who were out of work for more than 1 year (OR: 3.20, 95% CI: 2.94–3.49), out of work for less than 1 year (OR: 2.66, 95% CI: 2.41–2.93), homemaker (OR: 1.31, 95% CI: 1.21–1.42), student (OR: 1.32, 95% CI: 1.18–1.48), and unable to work (OR: 7.31, 95% CI: 6.89–7.75) had significantly higher odds of suffering from FMD. However, retired individuals had lower odds (OR: 0.84, 95% CI: 0.79–0.89) of reporting FMD. Those in the annual income categories higher than 15,000$ were significantly less likely to report FMD compared to adults having an annual income of less than 15,000$. The nonoverlapping confidence intervals indicated statistically significant decline in the odds of FMD with each higher income category; participants having an annual income of 50,000 $ or more showed the lowest odds of FMD (OR: 0.23, 95% CI: 0.22–0.25). Participants who were not married at the time of the survey had significantly higher odds of suffering from FMD, compared to those married at the time of the survey. Those who were separated from their partner (OR: 3.40, 95% CI: 3.10–3.72) had the highest odds followed by the divorced (OR: 2.13, 95% CI: 2.02–2.25) participants. 

Table 3 shows the association of tobacco and alcohol use, dietary practice, physical activity, chronic diseases, and BMI with FMD. Compared to people who were not diagnosed ever with any chronic health condition, individuals who ever had one or more conditions, such as high blood pressure, high cholesterol, heart disease, stroke, asthma, diabetes, and arthritis had higher odds of reporting FMD. The odds of developing FMD were nearly 10 times among people who had been ever diagnosed with all of the chronic conditions, compared to those without any of those conditions. Underweight (unadjusted OR: 1.74, 95% CI: 1.53, 1.98) and obese (unadjusted OR: 1.62, 95% CI: 1.54, 1.69) participants had significantly higher odds of having FMD compared with people in the normal range for BMI. 

### 3.3. Multivariable Analysis Results

Excluding the “don't know/not sure,” “refused,” and missing responses at random for all of the variables, a total of 380,637 participants were included in multivariable analysis. Underweight (AOR: 1.34, 95% CI: 1.13, 1.60) and obese (AOR: 1.13, 95% CI: 1.06, 1.20) participants were significantly more likely to report FMD (Table 4). Females had significantly higher odds of reporting FMD compared to males (AOR: 1.54; 95% CI: 1.46, 1.62). Non-Hispanic Blacks, non-Hispanic Asians, and Hispanics were less likely than non-Hispanic Whites to have FMD. Current or lifetime tobacco users (those who have smoked at least 100 cigarettes in their entire life) and binge drinkers were more likely to report FMD. Respondents involved in any kind of physical activity outside regular work were significantly less likely to suffer from mental distress. In comparison with participants who were employed for wages, others had higher adjusted odds of reporting FMD. Contrary to bivariate analysis results, retired persons had significantly higher odds of having FMD in multivariable analysis. The significantly reduced odds of FMD with increasing income also persisted even after controlling for all covariates. Divorced, separated, and never married people had higher adjusted odds of suffering from FMD, compared to those married when the survey took place. With each additional ever-diagnosed chronic health condition, there was a 33% increase in the odds of having FMD. 

### 3.4. Association of FMD with BMI Separately in Males and Females

As presented in Table 5, being out of work and being unable to work were significantly associated with FMD for both sexes. Divorced and separated males as well as females had higher odds of reporting FMD. Unlike women, widowed men had higher odds (AOR: 1.61, 95% CI: 1.35–1.93) of suffering from FMD compared to men who were married at the time of the survey. Higher income was associated with decreased odds of FMD for both genders. 

The overlapping confidence intervals indicate that adjusted ORs of having FMD were not significantly different between males and females for any of the categories of BMI (Table 5). Females, who were underweight (AOR: 1.33, 95% CI: 1.10, 1.60), overweight (AOR: 1.10, 95% CI: 1.03, 1.19), and obese (AOR: 1.21, 95% CI: 1.13, 1.31), had statistically significant higher odds of reporting FMD compared to females with normal BMI. For males, adjusted ORs of reporting FMD among underweight (AOR: 1.40, 95% CI: 0.98, 1.99), overweight (AOR: 0.97, 95% CI: 0.88–1.07), and obese (AOR: 1.05, 95% CI: 0.95–1.17) did not significantly differ from males with normal BMI. 

## 4. Discussion

### 4.1. Prevalence and Distribution of FMD

From this analysis, the prevalence of FMD (unweighted: 10.8%, weighted: 12%) among U.S. adults in 2011 was similar to that reported in the previous years [29, 46, 47]. The finding that females have a significantly higher risk of FMDs has also been consistent over the years [29]. Similar to previous findings [28, 29] people of all racial and ethnic backgrounds other than Asians were more likely to have FMD compared with non-Hispanic Whites. Interestingly, when all the other factors were controlled for, non-Hispanic Blacks and Hispanics were less likely to report FMD than Whites. In some earlier studies, Blacks and Hispanics were less likely to have depression and anxiety than Whites [48]. 

### 4.2. FMD and BMI in Males and Females Combined

From Table 4, people who were underweight and obese had higher adjusted odds of FMD compared to people with normal BMI. An analysis of data from the third (1988–1994) National Health and Nutrition Examination Survey (NHANES) found that severe obesity (BMI ≥ 40) was associated with depression [49]. Findings from the HUNT study [15] suggest an increase in the risk of depression with increase in BMI. However, the HUNT study was a prospective study, which evaluated an entirely different population, a county in Norway [15]. 

Unlike a previous finding [37], underweight people in this study had higher odds of FMD compared to those with normal BMI, even after adjusting for confounders, such as smoking, being unmarried, or unemployed [39–41]. The findings follow a pattern with people in both the lower and higher ranges of BMI having poorer mental health [50]. Certain specific mental problems (e.g., anxiety disorders) are often more common among underweight men [51]. 

In this analysis, consumption of fruits/fruit juice, vegetables, and beans decreased the risk of having FMD. This might be because healthy food items, such as fruits and vegetables, have a potential role in the prevention of mental health disorders [27]. Underweight people often suffer from malnutrition [52] and micronutrient deficiency, which are biological risk factors for poor mental health [53–55]. On the other hand, mental health problems, such as mood disorders and anorexia, may influence BMI [56, 57]. 

Tobacco use and lack of physical activity were significantly associated with FMD, similar to what is usually observed [9, 10]. The association of binge drinking with FMD in the present study corroborates with previous findings [58]. Similar to the findings by Zhao et al. [28, 50], being diagnosed with a chronic disease ever was associated with significantly higher odds of mental distress.

### 4.3. Gender-Specific Analysis of the Association between FMD and BMI

Gender-specific analysis showed that the adjusted ORs were not significantly different between the sexes, for any of the BMI categories. However, among women who were underweight, overweight, or obese, the odds of having FMD were significantly higher compared to women with normal BMI. Results of a previous study [49] are comparable to some extent, but it did not find any relationship between underweight and mental distress [49]. A 2005 report mentioned that a women-specific association may exist between obesity and depression [17]. Gender differences were not observed in all studies [28]. Anxiety and depression had a significantly higher prevalence among underweight, overweight, or obese women as well as underweight men in an analysis of 2006 BRFSS data [50]. In the Hunt Study [15] higher, but not lower, BMI was associated with an increased risk of depression at follow-up in both men and women. 

Obese women tend to internalize the ridicule and stigma experienced in public and from their own family members [21, 59], which might explain their mental distress. A distorted body image [22, 23], underlying anorexia nervosa, and dieting to lose weight [25] could influence the association between less than adequate BMI and poor mental health [23]. 

### 4.4. Limitations

This study has several limitations. All the variables were self-reported by the respondents and could be subject to recall bias. People may tend to underreport mental distress due to social-acceptability bias. Besides, the benchmark for having “good” or “not good” mental health can vary from person to person. Quality of sleep and its duration can affect both BMI [60] and mental health. Unfortunately, sleep related variables could not be included in this analysis, because of very few valid responses. Another potential confounder not taken into account is the intake of certain psychiatric medications, which can lead to weight gain [61]. The way in which some of the variables were combined for operational purposes (e.g., diet) was arbitrary and might not have been the best way to do so. For most of the questions, there were respondents who refused to answer or responded as “don't know/not sure.” These people, excluded from analysis, could be different in their behaviors, resulting in self-selection bias. However, a comparison of the characteristics of the sample between Tables 1 and 2 indicates that the percentages are fairly similar. Another drawback was that, after excluding all the missing values for all the variables, the sample size decreased considerably compared to bivariate analysis. This might partially be responsible for the differences in odds ratios between bivariate and multivariable analysis results. BMI is not always the most reliable indicator of body fat, and factors like the individual's waist circumference were not included in the survey [62, 63]. Mental health problems such as depression and anxiety, are not uncommon during pregnancy and it would be nice to look at the relationship of prepregnancy BMI, gestational gain in body weight, and mental distress among pregnant women [64, 65]. This was a cross-sectional survey; hence causality cannot be inferred. Also, there was no opportunity to evaluate the association of individual mental health disorders separately with the independent variables. Chronic health conditions have been grouped together, but some specific disorders, such as diabetes, are found to be associated with mental disorders, such as depression [66].

## 5. Conclusions

This study has used data from a very recent nationally representative sample. FMD, an indicator of Health-Related Quality of Life, indicates the assessment of a person about his or her own mental well-being [47]. A lot of confounding factors have been taken into account. The findings suggest that there could be a relationship between BMI and FMD independent of sociodemographic characteristics, risk-behaviors, lifestyle factors, and chronic diseases. Future research should explore longitudinal trend, whether abnormal BMI from an early age precedes mental distress, or vice versa. Measuring stigma and discrimination experienced by an overweight, obese, or underweight individual would be vital in understanding their role as potential mediators.

## Figures and Tables

**Table 1:** Distribution of the sample of adults in the United States, BRFSS 2011 by few sociodemographic factors, BMI, and FMD.

Variable	Categories	Number of respondents^a^	Weighted %	95% CI
Age category (years)	18–24	23069	12.88	12.63–13.14
	25–34	49621	17.65	17.40–17.89
	35–44	65487	17.56	17.34–17.79
	45–54	92177	18.87	18.66–19.09
	55–64	115569	15.46	15.29–15.63
	≥65	160544	17.58	17.42–17.74

Gender	Males	198812	48.73	48.44–49.02
	Females	307655	51.27	50.98–51.56

Race and ethnicity	Non-Hispanic White	396273	66.42	66.13–66.71
	Black non-Hispanics	41056	11.22	11.02–11.42
	Asian non-Hispanic	9492	3.93	3.78–4.08
	American Indian/Alaska Native non-Hispanic	7088	1.08	1.02–1.13
	Hispanic	38764	15.18	14.94–15.43
	Other race non-Hispanic	13794	2.17	2.08–2.25

Education	Did not graduate from high school	46423	15.42	15.17–15.67
	Graduated from high school	149387	29.22	28.96–29.49
	Attended college or technical school	136060	29.98	29.72–30.25
	Graduated from college or technical school	172669	25.38	25.16–25.60

Employment status	Employed for wages	207193	47.25	46.96–47.54
	Self-employed	40212	7.84	7.68–7.99
	Out of work for >1 year	17129	4.80	4.66–4.93
	Out of work for <1 year	13971	4.37	4.23–4.51
	Homemaker	35353	6.96	6.82–7.09
	Student	11435	5.83	5.65–6.02
	Retired	142070	16.39	16.23–16.55
	Unable to work	142070	6.56	6.43–6.70

BMI category	Normal	163451	34.86	34.57–35.14
	Underweight	8279	1.88	1.79–1.96
	Overweight	173661	35.85	35.56–36.13
	Obese	133116	27.42	27.16–27.68

FMD	Yes	53715	11.95	11.75–12.14
	No	442987	88.05	87.86–88.25

BMI: body mass index; CI: confidence interval; FMD: frequent mental distress; OR: odds ratio.

^a^The total number of respondents for the variables differs because of unequal number of missing values.

**Table 2:** Relationship of demographic characteristics with FMD among adults in the United States, BRFSS 2011.

Variable	Categories	Number of respondents^a^	%^c^	FMD			
				Number of respondents	%^d^	Unadjusted OR	95% CI
Age category^b^ (years)	18–24	22769	12.95	2788	12.24	1	
	25–34	48997	17.74	5920	12.08	1.10	1.01–1.21*
	35–44	64603	17.63	7827	12.12	1.13	1.04–1.24**
	45–54	90745	18.88	12461	13.73	1.23	1.13–1.34***
	55–64	113460	15.43	13837	12.2	1.10	1.01–1.19*
	≥65	156128	17.37	10882	6.97	0.61	0.56–0.67***

Gender^b^	Males	195315	48.76	17463	8.94	1	
	Females	301387	51.24	36252	12.03	1.39	1.34–1.44***

Race and ethnicity^b^	Non-Hispanic White	389117	66.58	39492	10.15	1	
	Black non-Hispanics	40073	11.18	5393	13.46	1.27	1.19–1.35***
	Asian non-Hispanic	9329	3.93	500	5.36	0.50	0.42–0.60***
	American Indian/Alaska Native non-Hispanic	6899	1.07	1152	16.70	1.72	1.51–1.96***
	Hispanic	37839	15.08	4867	12.86	1.10	1.04–1.17**
	Other race non-Hispanic	13445	2.16	2311	17.19	1.72	1.54–1.91***

Education^b^	Did not graduate from high school	44407	15.09	7953	17.91	1	
	Graduated from high school	146050	29.21	18043	12.35	0.62	0.58–0.66***
	Attended college or technical school	133821	30.12	15918	11.89	0.58	0.55–0.62***
	Graduated from college or technical school	170627	25.59	11601	6.80	0.31	0.29–0.33***

Employment status^b^	Employed for wages	204714	47.54	16393	8.01	1	
	Self-employed	39681	7.85	2958	7.45	1.01	0.93–1.10
	Out of work for >1 year	16683	4.77	3771	22.60	3.20	2.94–3.49***
	Out of work for <1 year	13709	4.36	2611	19.05	2.66	2.41–2.93***
	Homemaker	34526	6.94	3333	9.65	1.31	1.21–1.42***
	Student	11299	5.88	1393	12.33	1.32	1.18–1.48***
	Retired	138696	16.28	9369	6.76	0.84	0.79–0.89***
	Unable to work	34986	6.37	13590	38.84	7.31	6.89–7.75***

Annual income ($)	<15000	53554	13.51	12973	24.22	1	
	15000 to less than 25000	77697	18.67	11639	14.98	0.59	0.56–0.63***
	25000 to less than 35000	50913	11.47	5486	10.78	0.43	0.40–0.47***
	35000 to less than 50000	63685	13.92	5595	8.79	0.34	0.32–0.36***
	50000 or more	180314	42.43	10863	6.02	0.23	0.22–0.25***

Marital status	Currently married	264399	50.44	21435	8.11	1	
	Divorced	69630	10.27	11431	16.42	2.13	2.02–2.25***
	Widowed	67348	6.83	6643	9.86	1.31	1.23–1.40***
	Separated	10763	2.55	2699	25.08	3.40	3.10–3.72***
	Never married	69400	24.77	9423	13.58	1.56	1.48–1.65***
	A member of an unmarried couple	12672	5.13	1784	14.08	1.65	1.49–1.83***

CI: confidence interval; FMD: frequent mental distress; OR: odds ratio. *P < 0.05; **P < 0.01; ***P < 0.0001.

^a^The total number of respondents for the variables differs because of unequal number of missing values.

^b^The number of respondents within each category differs from that in Table 1, because all individuals with missing responses for the sociodemographic variable and FMD are excluded.

^c^Weighted percentage of respondents in each category out of the total number of respondents for that characteristic.

^d^Percentage with FMD among respondents within each category.

**Table 3:** Relationship of lifestyle factors, chronic disease, and BMI among adults in the United States, BRFSS 2011.

Variable	Categories	Number of respondents^a^	%^c^	FMD			
				Number of respondents	%^d^	Unadjusted OR	95% CI
Tobacco use	Never used tobacco or smoked <100 cigarettes in life	260343	53.82	20791	7.99	1	
	Everyday	68140	15.87	14418	21.16	2.98	2.84–3.14***
	Some of the days but not everyday	25669	6.63	4382	17.07	2.26	2.09–2.46***
	Previous user of smokeless tobacco or smoked <100 cigarettes in life	140339	23.68	13896	9.90	1.27	1.21–1.34***

Alcohol use	Did not use alcohol at all in the previous 30 days	224922	45.14	27909	12.41	1	
	Drank at least once in the past 30 days but not a heavy or binge drinker	167928	35.24	13558	8.07	0.64	0.61–0.67***
	Heavy drinker	7426	1.22	668	9.00	0.73	0.62–0.85***
	Binge drinker	60055	18.41	7190	11.97	0.96	0.90–1.01

Dietary practice (daily intake of fruits, fruit juice, vegetables, and beans)	None of them	1051	0.25	283	26.93	1	
	One of them	9824	2.13	1773	18.05	0.81	0.57–1.15
	Two of them	46658	9.52	6763	14.49	0.62	0.44–0.87**
	Three of them	166057	33.18	18767	11.30	0.47	0.33–0.65***
	All of them	254921	54.92	24041	9.43	0.38	0.27–0.54***

Physical activity in previous 30 days	No physical activity or exercise other than regular work	126867	25.52	20240	15.95	1	
	Met aerobic and strengthening guidelines	85231	19.72	6084	7.14	0.44	0.41–0.47***
	Met aerobic guidelines	151670	29.72	13009	8.58	0.53	0.50–0.55***
	Met strengthening guidelines	22798	6.12	2046	8.97	0.51	0.46–0.57***
	Met neither guideline but had physical activity	87991	18.92	9759	11.09	0.67	0.64–0.71***

Number of chronic diseases ever diagnosed^d^	None of them	157422	38.28	11082	7.04	1	
	One	130048	23.06	12432	9.56	1.52	1.44–1.61***
	Two	99824	13.90	11503	11.52	1.90	1.79–2.02***
	Three	65288	8.04	9381	14.37	2.38	2.23–2.54***
	Four	30769	3.43	5831	18.95	3.22	2.99–3.47***
	Five	10536	1.10	2599	24.67	4.26	3.85–4.72***
	Six	2447	0.22	751	30.69	5.86	4.93–6.98***
	Seven	368	0.03	136	36.96	9.76	6.53–14.61***

BMI category^b^	Normal	160465	34.88	14812	9.23	1	
	Underweight	8043	1.86	1235	15.35	1.74	1.53–1.98***
	Overweight	170601	35.85	15934	9.34	1.02	0.97–1.07
	Obese	130700	27.41	18846	14.42	1.62	1.54–1.69***

BMI: body mass index; CI: confidence interval; FMD: frequent mental distress; OR: odds ratio.

*P < 0.05; **P < 0.01; ***P < 0.0001.

^a^The total number of respondents for the variables differs because of unequal number of missing values.

^b^The number of respondents within each category differs from that in Table 1, because all individuals with missing responses for BMI and FMD are excluded.

^c^Weighted percentage of respondents in each category out of the total number of respondents for that characteristic.

^d^Percentage with FMD among respondents within each category.

**Table 4:** Adjusted odds ratios^a^ of having FMD among adults in the United States, BRFSS 2011 (*N* = 380637).

Variable^b^	Categories	Adjusted OR	95% CI
Age in years (ref: 18–24)	25–34	1.07	0.96–1.20
	35–44	1.04	0.93–1.17
	45–54	0.86	0.77–0.97*
	55–64	0.60	0.53–0.68***
	≥65	0.33	0.29–0.38***

Gender (ref: male)	Females	1.54	1.46–1.62***

Race and ethnicity (ref: non-Hispanic White)	Black non-Hispanics	0.84	0.78–0.92***
	Asian non-Hispanic	0.74	0.59–0.92**
	American Indian/Alaska Native non-Hispanic	1.07	0.90–1.27
	Hispanic	0.91	0.84–0.98*
	Other race non-Hispanic	1.26	1.11–1.43**

Education (ref: did not graduate from high school)	Graduated from high school	0.86	0.80–0.93**
	Attended college or technical school	0.95	0.87–1.03
	Graduated from college or technical school	0.79	0.72–0.87***

Employment status (ref: employed for wages)	Self-employed	1.10	1.00–1.21*
	Out of work for >1 year	2.14	1.93–2.37***
	Out of work for <1 year	1.99	1.77–2.23***
	Homemaker	1.17	1.06–1.28**
	Student	1.31	1.14–1.51**
	Retired	1.18	1.08–1.29**
	Unable to work	3.43	3.16–3.73***

Annual income in $ (ref: <15000)	15000 to less than 25000	0.77	0.72–0.83***
	25000 to less than 35000	0.72	0.66–0.79***
	35000 to less than 50000	0.63	0.57–0.69***
	50000 or more	0.53	0.48–0.58***

Marital status (ref: currently married)	Divorced	1.19	1.12–1.27***
	Widowed	1.06	0.97–1.16
	Separated	1.60	1.43–1.79***
	Never married	1.12	1.04–1.21**
	A member of an unmarried couple	1.16	1.02–1.31*

Tobacco use (ref: never used tobacco or smoked <100 cigarettes in life)	Everyday	1.92	1.80–2.04***
	Some of the days but not everyday	1.70	1.54–1.87***
	Previous user of smokeless tobacco or smoked <100 cigarettes in life	1.22	1.15–1.29***

Alcohol use (ref: did not use alcohol at all in the previous 30 days)	Drank at least once in the past 30 days but not a heavy or binge drinker	0.98	0.92–1.03
	Heavy drinker	1.08	0.89–1.31
	Binge drinker	1.17	1.09–1.25***

Dietary practice^c^		0.92	0.90–0.95***

Physical activity in previous 30 days (ref: no physical activity or exercise other than regular work)	Met aerobic and strengthening guidelines	0.68	0.63–0.73***
	Met aerobic guidelines	0.71	0.67–0.76***
	Met strengthening guidelines	0.78	0.70–0.88***
	Met neither guideline but had physical activity	0.81	0.76–0.86***

Number of chronic conditions ever diagnosed^c^		1.33	1.31–1.36***

BMI category (ref: normal)	Underweight	1.34	1.13–1.60**
	Overweight	1.03	0.97–1.10
	Obese	1.13	1.06–1.20***

BMI: body mass index; CI: confidence interval; FMD: frequent mental distress; OR: odds ratio.

*P < 0.05; **P < 0.01; ***P < 0.0001.

^a^OR for each variable is adjusted for all other covariates.

^b^The reference category for each variable is specified within parenthesis.

^c^Introduced in the multivariable model as continuous variable.

**Table 5:** Adjusted odds ratios^a^ of having FMD among adults in the United States, stratified by gender, BRFSS 2011.

Variable^b^	Categories	Females (*N* = 221623)		Males (*N* = 159014)	
		Adjusted OR	95% CI	Adjusted OR	95% CI
Age category (years) (ref: 18–24)	25–34	1.06	0.91–1.23	1.11	0.94–1.31
	35–44	1.04	0.89–1.21	1.06	0.89–1.26
	45–54	0.88	0.76–1.03	0.83	0.70–0.99*
	55–64	0.61	0.52–0.71***	0.60	0.50–0.73***
	≥65	0.34	0.28–0.40***	0.34	0.27–0.42***

Race and ethnicity (ref: non-Hispanic White)	Black non-Hispanic	0.81	0.73–0.89***	0.88	0.77–1.01
	Asian non-Hispanic	0.77	0.57–1.04	0.72	0.53–0.99*
	American Indian/Alaska Native non-Hispanic	0.93	0.75–1.15	1.20	0.91–1.57
	Hispanic	0.90	0.81–0.99*	0.92	0.81–1.04
	Other race non-Hispanic	1.15	0.97–1.35	1.38	1.13–1.68**

Education (ref: did not graduate from high school)	Graduated from high school	0.89	0.81–0.99*	0.84	0.74–0.95**
	Attended college or technical school	0.99	0.89–1.10	0.90	0.79–1.03
	Graduated from college or technical school	0.82	0.73–0.92**	0.75	0.65–0.87***

Employment status (ref: employed for wages)	Self-employed	1.10	0.96–1.24	1.14	1.00–1.29
	Out of work for >1 year	1.95	1.72–2.21***	2.36	2.01–2.78***
	Out of work for <1 year	1.83	1.57–2.12***	2.15	1.82–2.56***
	Homemaker	1.10	1.00–1.22*	—	
	Student	1.41	1.18–1.67***	1.14	0.88–1.46
	Retired	1.03	0.94–1.14	1.33	1.14–1.56**
	Unable to work	3.23	2.92–3.57***	3.66	3.19–4.20***

Annual income ($) (ref: <15000)	15000 to less than 25000	0.75	0.69–0.82***	0.80	0.71–0.90**
	25000 to less than 35000	0.71	0.63–0.79***	0.74	0.64–0.86***
	35000 to less than 50000	0.59	0.53–0.66***	0.67	0.58–0.78***
	50000 or more	0.53	0.48–0.60***	0.53	0.46–0.61***

Marital status (ref: currently married)	Divorced	1.16	1.08–1.26**	1.23	1.11–1.37**
	Widowed	0.95	0.86–1.05	1.61	1.35–1.93***
	Separated	1.58	1.38–1.82***	1.61	1.33–1.95***
	Never married	1.11	1.00–1.22*	1.13	1.01–1.26*
	A member of an unmarried couple	1.18	1.02–1.37*	1.13	0.92–1.38

Tobacco use (ref: never used tobacco or smoked <100 cigarettes in life)	Everyday	2.02	1.87–2.18***	1.77	1.60–1.97***
	Some of the days but not everyday	1.71	1.52–1.92***	1.66	1.43–1.93***
	Previous user of smokeless tobacco or smoked <100 cigarettes in life	1.20	1.12–1.29***	1.20	1.08–1.32**

Alcohol use (ref: not used alcohol at all in the previous 30 days)	Drank at least once in the past 30 days but not a heavy or binge drinker	1.05	0.98–1.12	0.88	0.80–0.96**
	Heavy drinker	1.09	0.88–1.34	1.06	0.70–1.61
	Binge drinker	1.27	1.16–1.40***	1.08	0.98–1.19

Dietary practice^c^		0.94	0.90–0.98**	0.91	0.87–0.96**

Physical activity in previous 30 days (ref: no physical activity or exercise other than regular work)	Met aerobic and strengthening guidelines	0.65	0.59–0.72***	0.71	0.63–0.80***
	Met aerobic guidelines	0.68	0.63–0.73***	0.75	0.68–0.83***
	Met strengthening guidelines	0.72	0.62–0.83***	0.86	0.72–1.02
	Met neither guideline but had physical activity	0.78	0.72–0.84***	0.85	0.75–0.95**

Number of chronic conditions ever diagnosed^c^		1.34	1.31–1.37***	1.33	1.29–1.38***

BMI category (ref: normal)	Underweight	1.33	1.10–1.60**	1.40	0.98–1.99
	Overweight	1.10	1.03–1.19**	0.97	0.88–1.07
	Obese	1.21	1.13–1.31***	1.05	0.95–1.17

BMI: body mass index; CI: confidence interval; FMD: frequent mental distress; OR: odds ratio.

*P < 0.05; **P < 0.01; ***P < 0.0001.

^a^OR for each variable is adjusted for all other covariates.

^b^The reference category for each variable is specified within parenthesis.

^c^Introduced in the multivariable model as continuous variable.
